# Effect of Ketamine Supplementation in Axillary Plexus Blockade: A Comparative Study

**DOI:** 10.7759/cureus.99919

**Published:** 2025-12-23

**Authors:** Demetra Solomos, Aggeliki Bairaktari, Theodoros Xanthos, Kassiani Theodoraki

**Affiliations:** 1 Department of Anesthesiology, KAT Hospital, Athens, GRC; 2 Department of Midwifery, School of Health and Care Sciences, University of West Attica, Athens, GRC; 3 Department of Anesthesiology, Aretaion University Hospital of Athens, Rhodes, GRC

**Keywords:** axillary block, brachial plexus, ketamine, nrs score, onset time, postoperative analgesia, rebound pain, ropivacaine

## Abstract

Introduction

Axillary brachial plexus block is a widely used regional anesthesia technique for below-elbow surgeries. The use of adjuvant medications can influence block characteristics and postoperative pain experience. This randomized, prospective, single-blind comparative study aimed to evaluate whether ketamine, administered intravenously or regionally with ropivacaine, affects postoperative analgesia, rebound pain, and the onset time of sensory and motor block.

Methods

A randomized, prospective, single-blind comparative study was performed in patients undergoing ultrasound-guided axillary brachial plexus block for below-elbow surgery. Participants were assigned to three groups: group 1 (control, ropivacaine alone), group 2 (intravenous ketamine plus ropivacaine), or group 3 (regional ketamine plus ropivacaine). Onset of sensory and motor block, postoperative pain intensity using the numerical rating scale (NRS), need for rescue analgesia, and adverse effects during the first 24 hours were recorded and compared.

Results

The study included 81 patients (49 men and 32 women) with a mean age of 44.27±17.61 years (range: 16-81 years). Among them, 28 patients were in group 1, 27 patients in group 2, and 26 patients in group 3. Univariate analysis showed that both regional and intravenous ketamine administration were associated with faster onset of blockade (p<0.0005) and significantly lower NRS scores than the local anesthetic alone, at 16, 20, and 24 hours after the end of surgery (p=0.049, p=0.009, and p=0.006, respectively). No statistically significant difference was observed in motor block scores (p=0.329) and postoperative opioid (tramadol) intake (p=0.888) among the three groups.

Conclusions

Intravenous and regional addition of ketamine to ropivacaine solution improved postoperative analgesia equally by attenuating rebound pain and reducing the onset time of sensory and motor block. More high-quality studies are needed to fully elucidate the role of ketamine in axillary brachial plexus block.

## Introduction

Brachial plexus blockade is an effective method for providing regional anesthesia to the upper limb from the shoulder to the fingertips, by blocking the transmission of nerve impulses in the brachial plexus [1]. Axillary brachial plexus block has become popular for below-elbow surgical procedures, as it attenuates postoperative pain, provides satisfactory anesthesia as well as postoperative analgesia, and is well tolerated, having a low incidence of complications [2]. Other benefits of axillary brachial plexus blockade relate to the reduction in the need for general anesthesia and associated adverse effects such as respiratory depression, decrease of cardiac output, and central nervous system depression. Moreover, axillary plexus blockade provides superior postoperative pain management compared to general anesthesia, diminishing the need for postoperative opioid administration. An additional benefit of using a regional anesthesia technique is the lower procedure cost [3].

Increasing the efficacy and duration of regional anesthesia in upper limb surgery is crucial for the extension of postoperative analgesia, allowing faster rehabilitation of patients [4]. Ropivacaine is widely used in axillary brachial plexus block for its prolonged action. Ultrasound-guided blockades may be precise and reduce the block performance time [5]. However, further prolongation of the blockade by increasing the dose of local anesthetic is not always feasible due to local anesthetic-associated potential toxicity. Therefore, as an alternative, there is a plethora of additives available, used for increasing local anesthetic efficacy and duration, while maintaining safety. These adjuvants include opioids, alpha-2 agonist, anti-inflammatory agents, steroids, midazolam, and magnesium [6-8], which have local vasoconstrictive action or act directly on peripheral nerves through anti-inflammatory action [9].

Ketamine is an N-methyl-D-aspartate (NMDA) receptor antagonist, used primarily for the induction and maintenance of anesthesia and occasionally for the treatment of neuropathic pain and depression. By blocking the NMDA receptor, ketamine is able to prevent the transmission of pain signals to the brain, thus providing effective pain relief. The distinguishing features of anesthesia achieved by ketamine administration are preservation of respiration and airway reflexes, stimulated cardiac function with increased blood pressure, and moderate bronchodilation [10]. Recently, there has been increasing interest in the use of ketamine for peripheral nerve blockade, specifically for brachial plexus blockade [11]. However, the exact mechanism through which ketamine could manifest analgesic action via the perineural route remains unclear. It is hypothesized that this effect could be due to the voltage-dependent blockade of the NMDA receptor, which abolishes hypersensitivity and prevents central sensitization [12]. It has also been suggested that ketamine may enhance the binding capacity of local anesthetics to albumin and alpha-1-acid glycoprotein [12].

The primary objective of the study was to evaluate the effects of adding ketamine to ropivacaine, administered either intravenously or regionally, on the intensity of postoperative pain and the incidence of rebound pain. The secondary objectives were to assess the onset and duration of the axillary block.

## Materials and methods

This three-arm prospective, randomized, single-blind study was conducted in patients undergoing forearm, wrist, or hand surgery with the method of axillary brachial plexus blockade. Patients and the data analyst were blinded, while the block-performing anesthesiologist was not. After Institutional Review Board approval of the study, all individuals were fully informed of its purpose and signed the relevant consent form.

Inclusion criteria were patients aged 16 to 80 years old, with body mass index (BMI) 20-35 kg/m^2^, American Society of Anesthesiologists (ASA) score 1-3, and subjected to below-elbow surgery under ultrasound-guided axillary brachial plexus blockade. Patients with an allergy to local anesthetics, peripheral neuropathy, psychiatric, liver, or renal disease, along with a history of gastrointestinal bleeding, were excluded from the study.

Eligible participants were randomized with the help of a computer-generated sequence of random numbers into three groups as follows: group 1 (control group) received 30 ml of ropivacaine 0.5% regionally, group 2 (intravenous ketamine group) received 30 ml of ropivacaine 0.5% regionally and a bolus dose of 30 mg of ketamine intravenously and group 3 (regional ketamine group) received 30 ml of ropivacaine 0.5% along with 30 mg of ketamine regionally. For regional administration, 30 mg of ketamine was diluted to a volume of 1 ml and added directly to the local anesthetic solution prior to injection [11]. Patients were blinded to group allocation, along with the investigator analyzing the final data of the study, to whom group allocation was concealed.

After arrival in the operating room, patients were placed in a supine position, with the arm in 90°abduction and the elbow in 90° flexion, with the shoulder in external rotation and the whole arm next to the patient's head. Ten minutes before axillary blockade, all patients received 40 mg omeprazole intravenously, 4 mg ondasetron, and a midazolam solution of 0.01-0.1 mg/kg for mild sedation. Under ultrasound guidance (General Electric Venue Fit,** **GE HealthCare, Chicago, IL, USA), with linear high frequency (8-13 MHz), 10 ml of the local anesthetic ropivacaine (Ropivacain HCl B. Braun, B. Braun Melsungen AG, Melsungen, Germany,​​​​​** **7.5 mg/ml, LOT 23243010) was administered dorsally to the axillary artery. Then, the needle (Pajunk SonoPlex II, PAJUNK GmbH Medizintechnologie, Geisingen, Germany, 22G×50mm) was redirected to the median and ulnar nerve, where another 12 ml was administered. Finally, the needle was withdrawn back into the biceps and redirected to the musculocutaneous nerve, and when the needle was adjacent to the nerve, a further 8 ml of local anesthetic was administered.

Sensory blockade was evaluated by the pinprick test in the area of distribution of the four nerves (median, radial, ulnar, and musculocutaneous) [13]. For the assessment of motor blockade, a three-level scale was used, with 0 representing complete paralysis, 1 indicating partial paralysis, and 2 indicating maintenance of the full range of motion. The degree of sensory and motor blockade of the axillary block was evaluated using the above two techniques and recorded every two minutes by the anesthesiologist performing the blockade, before the start of the operation.

Pain intensity was evaluated with the numerical rating scale (NRS or NRS-11), which is an 11-point scale graded from 0 (no pain) to 10 (worst possible pain). The principle and use of the NRS score were explained to all patients preoperatively. To facilitate the process of pain intensity self-reporting, the image of a 10 cm ruler was provided in each patient's questionnaire. According to the study protocol, in case the patient complained of pain (NRS≥4) or unpleasant sensation in the operated limb intraoperatively, the block was considered unsuccessful, and general anesthesia was administered to the patient. These patients were then excluded from further analysis.

Postoperatively, all patients received systemic intravenous paracetamol 1 g three times a day, dexketoprofen 50 mg twice a day, and ondansetron 4 mg twice a day. In case of patient perceived pain (NRS≥4), intravenous tramadol 100 mg was administered (rescue analgesia) up to a maximum of 300 mg per day. All patients were monitored postoperatively for approximately 24 hours, when they were discharged, and no contact with them took place after this period.

Chronologically, from the start to the end of the study period of each patient, the following data were recorded and compared for all three groups, including patient demographic characteristics (age, gender, BMI) and time of onset of sensory and motor block. Onset time of sensory or motor blockade was defined as the time period between the end of injection and the absence of pain around the affected area or complete motor paralysis, respectively [4]. Moreover, the duration of surgery in minutes (initial skin incision to final suture) was recorded along with pain intensity/rebound pain assessed with the NRS, graded from a minimum of 0 to a maximum of 10. Recording was performed at 0, 4, 8, 16, 20, and 24 hours after the end of the operation. Adverse effects like nausea, vomiting, and hallucinations that the patient experienced from the start of the blockade up to the next 24 hours were compared among groups, along with the total dose of tramadol administered to the patient postoperatively. Rebound pain was defined as the transition from well-controlled mild pain (0≤NRS≤3) to an NRS≥4 within 24 hours of block performance. Two subcategories of rebound pain were further distinguished: moderate (4≤NRS≤6) or severe rebound pain (7≤NRS≤10).

Statistical analysis

The enhancing anesthetic/analgesic effects of the regional administration of ketamine have been well established [10-12]. Based on a priori power analysis, it was estimated that at least 16 patients per group were needed to detect a 35% change in duration and strength of analgesia to achieve a statistical power of 80% at significance 0.05. We aimed for 30 patients per group to adjust for potential dropouts.

Quantitative variables were expressed as mean and standard deviation (SD) or median and interquartile range (IQR). Frequencies (n) and the corresponding percentages (%) were used for depiction of qualitative variables. Testing for normality of the measurements was done using the Kolmogorov-Smirnov test. Comparison of intraoperative quantitative variables among the groups was performed using a t-test for independent samples and one-way ANOVA with the Bonferroni test for pairwise comparisons, while the Kruskal-Wallis and Mann-Whitney tests were used in case the data were not normally distributed. The chi-square test was used for comparisons of qualitative variables.

All tests were two-sided, and a value of α=0.05 was set as the level of significance. All statistical analyses were performed with the statistical package SPSS version 21.00 (IBM Inc., Armonk, USA).

## Results

**Figure 1:**
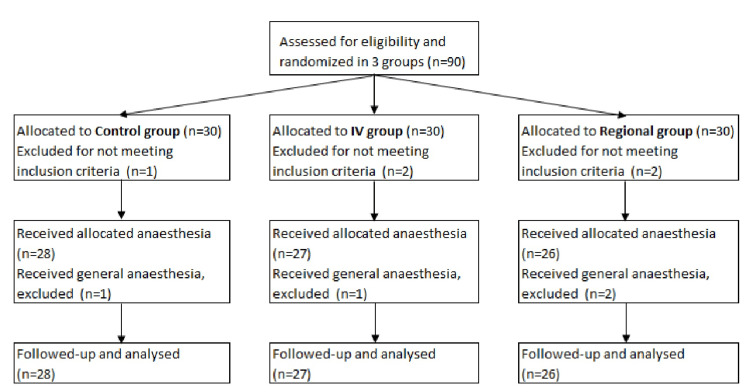
Consort diagram of the study

Ninety patients in total were initially enrolled in the study and assigned to three equal groups. Five of them were subsequently excluded because they did not meet the inclusion criteria mentioned above. Additionally, four more patients were removed from the study, as general anesthesia had to be administered to them due to a failed block. Therefore, the final numbers of patients analyzed and the anesthesia received were as follows: group 1 (control group) n=28, received 30 ml of ropivacaine 0.5% regionally, group 2 (intravenous ketamine group) n=27, received 30 ml of ropivacaine 0.5% regionally and a bolus dose of 30 mg of ketamine intravenously and group 3 (regional ketamine group) n=26, received 30 ml of ropivacaine 0.5% along with 30 mg of ketamine regionally (Figure 1).

Patient characteristics of the study population are summarized in Table 1. Eighty-one patients (49 men, 32 women) with a mean age of 44.37±17.64 years (range: 16-81 years) were finally analyzed. Τhere was no significant difference associated with the age, gender, BMI, and duration of surgery among the three groups (for all comparisons, p>0.05).

**Table 1:** Characteristics of the study sample Continuous variables are presented as mean±SD. Age, BMI, and duration of surgery among groups were compared with a one-way ANOVA test.

Variables		Control group (n=28)	Intravenous ketamine group (n=27)	Regional ketamine group (n=26)	p-value	F-value
Age (years)		40.46±20.69	48.44±14.08	44.35±17.09	0.248	1.41
BMI (kg/m^2^)		26.13±4.44	27.26±3.20	26.08±3.33	0.420	1.15
Duration of surgery (min)		72.50±28.82	71.11±26.40	80.04±33.09	0.500	0.70

**Figure 2:**
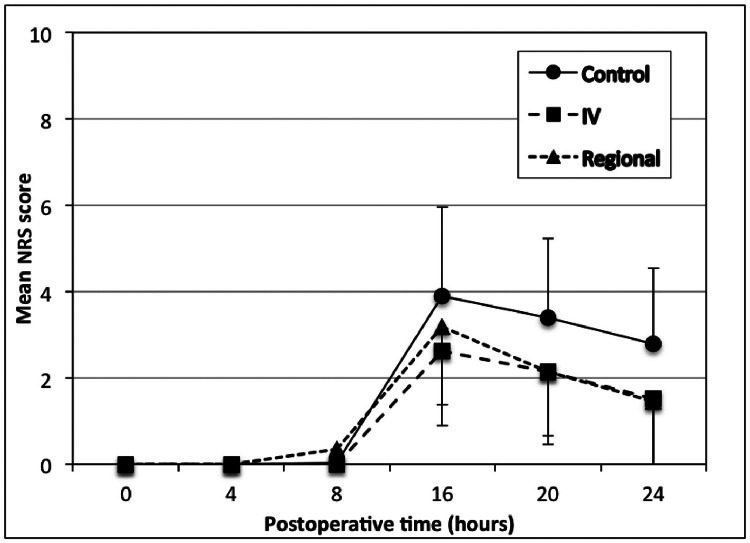
Comparison of NRS pain scores among the three groups at 0, 4, 8, 16, 20 and 24 hours postoperatively following the axillary brachial plexus blockade NRS - numerical rating scale

Regarding the primary outcome of postoperative analgesia, Table 2 and Figure 2 show the mean and standard deviation of the NRS pain scores of the patients at 0, 4, 8, 16, 20, and 24 hours after the operation. No differences were observed in the NRS score between the groups at time 0 (p=1.000), at 4 hours (p=1.000), and at 8 hours (p=0.182).

**Table 2:** Comparison of the NRS pain scores among the three groups at 0, 4, 8, 16, 20 and 24 hours The variable is presented as mean±SD. NRS pain scores among groups were compared with a one-way ANOVA test with Bonferroni correction. NRS - numerical rating scale

NRS	Control group (n=28)	Intravenous ketamine group (n=27)	Regional ketamine group (n=26)	p-value	F-value
NRS 0 h	0.0±0.0	0.0±0.0	0.0±0.0	1.000	-
NRS 4 h	0.0±0.0	0.0±0.0	0.0±0.0	1.000	-
NRS 8 h	0.04±0.19	0.0±0.0	0.35±1.29	0.182	1.744
NRS 16 h	3.89±2.06	2.63±1.74	3.19±1.81	0.049	3.127
NRS 20 h	3.39±1.83	2.15±1.49	2.15±1.69	0.009	5.021
NRS 24 h	2.79±1.75	1.52±1.60	1.46±1.63	0.006	5.559

At 16 hours, a statistically significant difference in the NRS pain score (p=0.049) was observed among the three groups. When examined pairwise, the intravenous ketamine group presented a lower NRS pain score than the control group (p=0.044). There was no statistically significant difference between the regional ketamine and the control groups (p=0.524), and between the regional ketamine and intravenous ketamine groups (p=0.836).

At 20 hours, there was a statistically significant difference in the means of the NRS pain score (p=0.009) among the three groups. In the pairwise testing, the intravenous ketamine and regional ketamine groups presented a lower NRS score than the control group (p=0.022 and p=0.025, respectively), while there was no difference between the regional ketamine and intravenous ketamine groups (p=1.000).

At 24 hours, there was a statistically significant difference in the means of the NRS pain score (p=0.006) among the three groups. In the pairwise testing, the intravenous ketamine and regional ketamine groups presented a lower NRS score than the control group (p=0.018 and p=0.014, respectively), while there was no difference between the regional ketamine and intravenous ketamine groups (p=1.000).

Table 3 summarizes the above pairwise statistical testing results for 16, 20, and 24 hours postoperatively, supplemented with the corresponding 95% confidence intervals of the differences of the means (μG1, μG2, μG3) of the three groups. It can be safely stated that, as set out in the sample size estimation, the one NRS pain score point difference of the means of groups 2 and 3 with respect to group 1 has been achieved at 16, 20, and 24 hours postoperatively. No such difference exists in the average pain scores of Groups 2 and 3 (third column), which, in the sense of postoperative analgesia, means that they perform equally well.

**Table 3:** Comparison of the p-values and the 95% confidence intervals of the difference in means of the three groups at 16, 20 and 24 hours postoperatively (using t-test for independent samples) NRS - numerical rating scale

NRS	Control - intravenous groups μ_G1_–μ_G2_		Control - regional groups μ_G1_–μ_G3_		Regional - intravenous groups μ_G3_–μ_G2_	
	p-value	95% CI	p-value	95% CI	p-value	95% CI
NRS 16 h	0.044	[0.23, 2.29]	0.524	[-0.36, 1.76]	0.836	[-0.42, 1.54]
NRS 20 h	0.022	[0.34, 2.14]	0.025	[0.28, 2.20]	1.000	[-0.88, 0.88]
NRS 24 h	0.018	[0.36, 2.18]	0.014	[0.41, 2.25]	1.000	[-0.95, 0.83]

**Figure 3:**
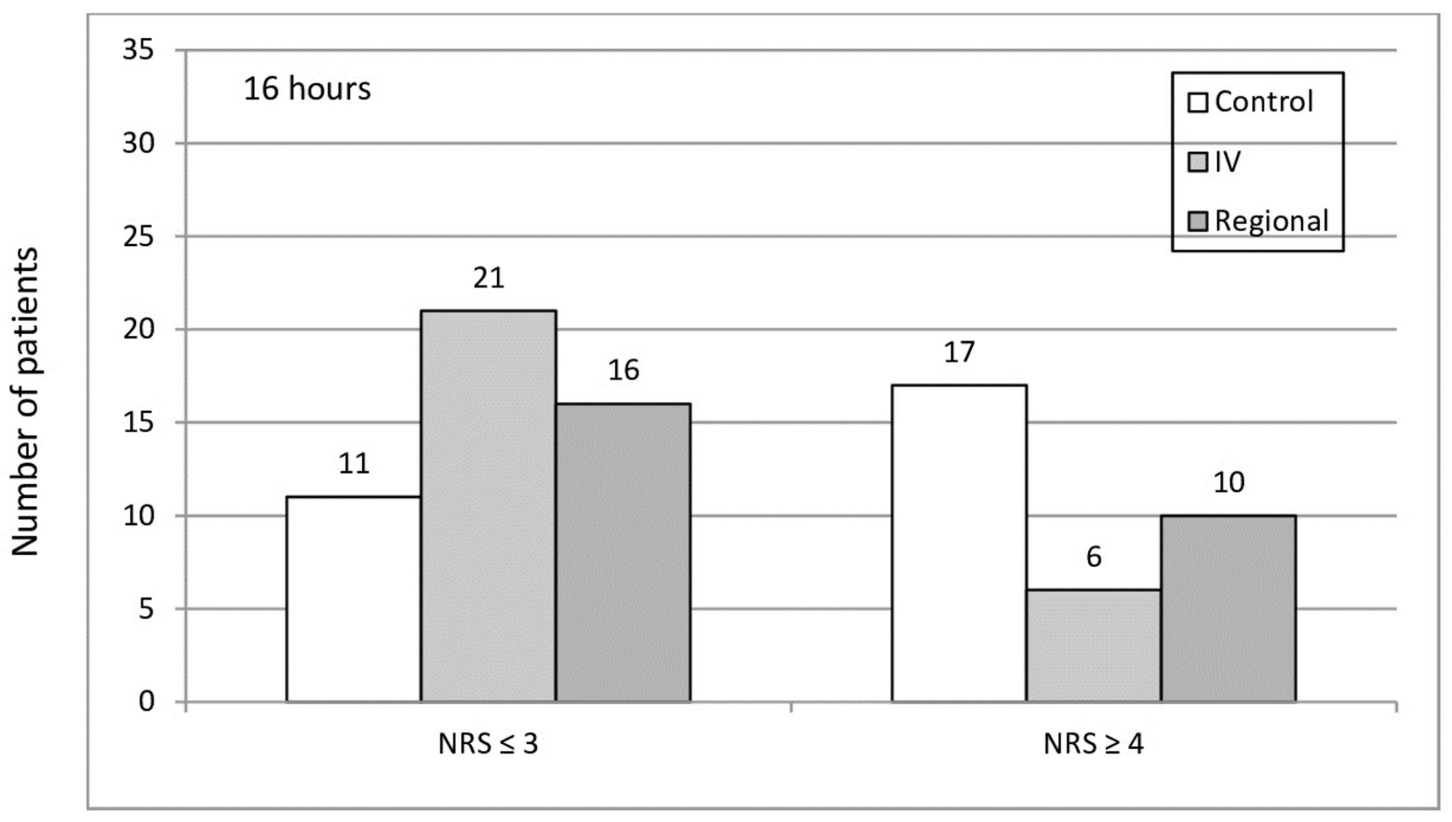
Distribution of pain scores at 16 hours postoperatively between the mild postoperative pain class (NRS≤3) and the moderate/severe rebound pain class (NRS≥4) for the three groups NRS - numerical rating scale

Table 4 and Figure 3 present the incidence of rebound pain at 16 hours after the operation, when the peak NRS pain scores were reported by the patients. Chi-square testing for the three study groups showed that they had different behavior with respect to the manifestation of rebound pain (p=0.014), with the intravenous and regional ketamine groups demonstrating a considerably lower incidence of rebound pain as compared to the control group.

**Table 4:** The comparison of the severity of postoperative pain in the patients of the three study groups at 16 hours postoperatively, when peak pain scores were reported NRS - numerical rating scale

Pain severity class	Control group (n=28)	Intravenous ketamine group (n=27)	Regional ketamine group (n=26)
Mild (0≤NRS≤3)	11	21	16
Moderate (4≤NRS≤6)	16	5	9
Severe (7≤NRS≤10)	1	1	1

**Figure 4:**
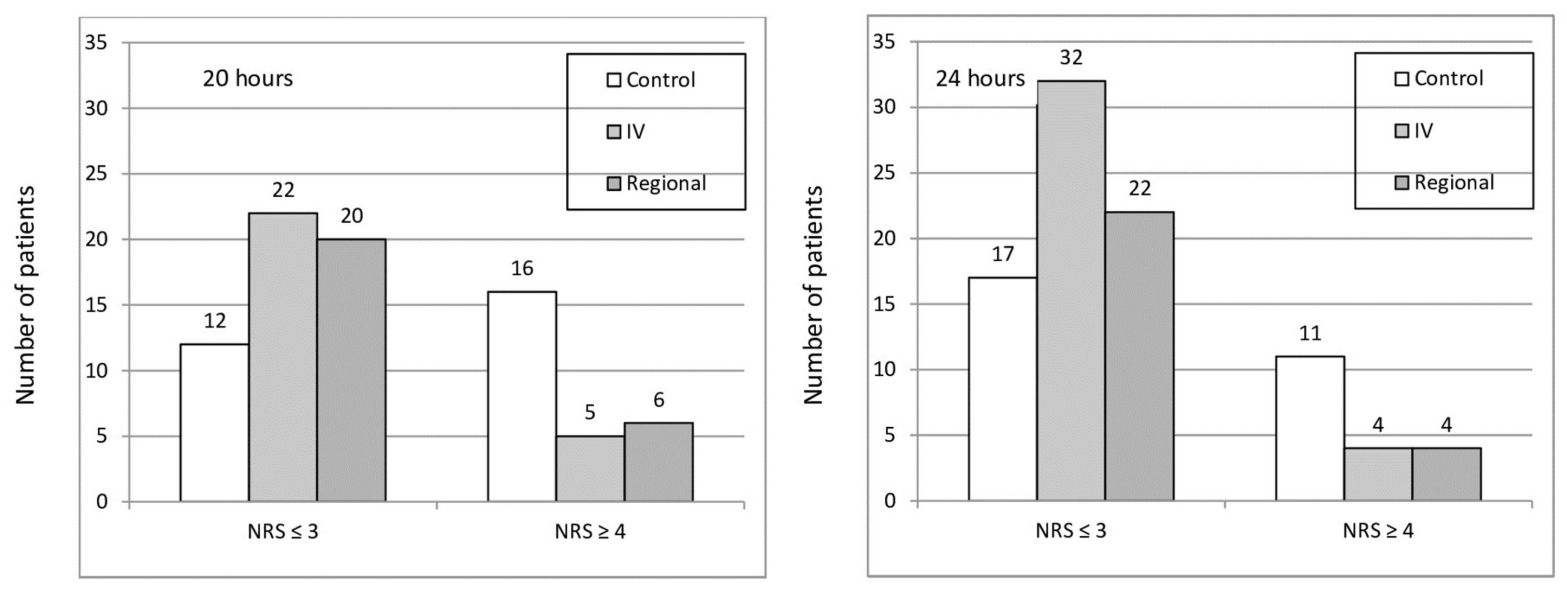
Distribution of pain scores at 20 and 24 hours postoperatively between the mild postoperative pain class (NRS≤3) and the moderate/severe rebound pain class (NRS≥4) for the three groups NRS - numerical rating scale

Similarly, Figure 4 presents the incidence of rebound pain at 20 and 24 hours postoperatively. Clearly, the scores of the intravenous and regional ketamine groups populate more and more the mild pain class (NRS≤3) and less the moderate/severe rebound pain class (NRS≥4).

After establishing the primary outcomes, the secondary objectives of the study are investigated. As shown in Table 5, there were no significant differences in the incidence of intraoperative dizziness, nausea, and vomiting. However, it was observed that the intravenous administration of ketamine was associated with a significantly higher number of hallucination episodes in comparison to the control group (p=0.003) and the regional ketamine group (p=0.007).

**Table 5:** Comparison of side effects among groups The chi-square test was used for comparisons of the frequencies of adverse events.

Side effects	Control group (n=28)	Intravenous ketamine group (n=27)	Regional ketamine group (n=26)	p-value	X^2^-value
Dizziness, n (%)	0 (0%)	1 (3.7%)	0 (0%)	0.363	2.025
Nausea, n (%)	0 (0%)	0 (0%)	1 (3.8%)	0.343	2.142
Vomiting, n (%)	0 (0%)	0 (0%)	0 (0%)	1.000	-
Hallucination episodes, n (%)	0 (0%)	9 (33.3%)	0 (0%)	<0.0005	20.250

Onset time of sensory and motor block was significantly lower in the intravenous ketamine group (15.19±2.45 min) and the regional ketamine group (16.77±2.57 min) in comparison to the control group (19.04±3.44) (p<0.0005). No difference was observed between intravenous and regional ketamine administration in this respect (p=0.143).

As shown in Table 6, no difference was detected in complete motor blockade and in postoperative tramadol administration among the three groups (p=0.33 and p=0.89, respectively). Out of the 81 patients, the motor blockade was complete in 69 (85.2%). Only eight patients (9.8%) required the administration of 100 mg of tramadol, and no more than 100 mg of tramadol were administered to any patient.

**Table 6:** Comparison of motor block score and of tramadol intake (rescue analgesia) among the three groups The chi-square test was used for comparisons of motor blockade and tramadol administration.

Variables		Control group (n=28)	Intravenous ketamine group (n=27)	Regional ketamine group (n=26)	p-value	X^2^-value
Complete motor blockade	Yes, n (%)	24 (85.7%)	21 (77.8%)	24 (92.3%)	0.329	2.225
	No, n (%)	4 (14.3%)	6 (22.2%)	2 (7.7%)		
100 mg tramadol administration	Yes, n (%)	3 (10.7%)	2 (7.4%)	3 (11.5%)	0.888	0.288
	No, n (%)	25 (89.3%)	25 (92.6%)	23 (88.5%)		

## Discussion

The present study aimed to investigate the effects of ketamine addition to the ropivacaine solution for performing axillary brachial plexus block, particularly in regard to its postoperative analgesic effects. Concerning this primary objective, it was observed that both regional and intravenous administration of ketamine provided a stronger analgesic effect, as quantified by the rebound pain, than ropivacaine alone, at 16, 20, and 24 hours after the end of the block. Ketamine administration either regionally or intravenously was also associated with a faster onset of nerve block, while intravenous ketamine was associated with a significantly higher incidence of hallucinations. No statistically significant difference was found in complete motor blockade scores and postoperative opioid (tramadol) intake.

Four similar prospective, randomized, double-blind studies were identified in the literature that evaluated the role of ketamine addition either via regional or intravenous administration to the local anesthetic for axillary brachial plexus block [14-17]. The addition of 30-50 mg ketamine to lidocaine or ropivacaine was found to have inferior analgesic properties compared to the addition of fentanyl [14], tramadol [15], and dexamethasone [17]. Moreover, in a study by Touil et al., intravenous ketamine administration at a dose of 0.3 mg/kg had no significant benefits in postoperative analgesia as compared with placebo and had similar effects to placebo in resolving rebound pain [16]. In contrast to the aforementioned studies, our study showed that the administration of ketamine, both regionally and intravenously, was associated with a faster onset of block and reduced postoperative pain at 16 to 24 hours after axillary brachial plexus block. The observed analgesic benefit during the 16-24 hour postoperative window is clinically relevant, as this period commonly coincides with hospital discharge or the first postoperative night in ambulatory or short-stay surgery. Effective pain control during this timeframe may reduce unplanned healthcare utilization, improve patient comfort, and mitigate rebound pain following block resolution. It should be noted, however, that the aim of the present study was to focus on the addition or not of ketamine as an adjuvant compared to the local anesthetic alone and not on its comparison with other adjuvants, which, as mentioned above, have exhibited better analgesic effects. An additional novelty of our study is the fact that we investigated the influence of the administration route (intravenously versus regionally) on the overall analgesic effect, a topic that has scarcely been investigated in the literature.

Rebound pain after the resolution of a peripheral nerve block is an important issue, particularly for outpatient surgery. Rebound pain is the relatively rapid increase in the severity of pain after resolution of the block [15,16]. As noted, the study by Touil et al. showed that intravenous ketamine administration did not have a favorable effect on the frequency and intensity of rebound pain after axillary brachial plexus block [16]. On the contrary, there are other studies suggesting that adjuvants, including ketamine, help in moderating rebound pain [18]. Our study points in this direction too, since it was observed that the incidence of moderate and severe rebound pain was lower when ketamine was supplemented with the local anesthetic, either regionally or intravenously. It should also be noted that the maximum NRS score reported (NRS=8) was in a patient in the control group. Although ketamine was administered regionally in one study group, partial systemic absorption cannot be excluded. Therefore, the observed reduction in rebound pain may reflect a combination of local peripheral effects and low-level systemic ketamine activity. This possibility should be considered when interpreting the analgesic benefits observed in the regional administration group.

Apart from the axillary plexus blockade, ketamine use has also been tested in interscalene brachial plexus blockade. Lee et al. found no significant effect of the addition of 30 mg ketamine, either regionally or intravenously, in interscalene brachial plexus blockade, on the onset time and duration of sensory or motor blockade. Authors found a higher incidence of adverse events in the intravenous group (94% vs 44%) [19]. In our study, these parameters were found to show some improvement. The study by Lee et al. provides a basis of comparison of the effectiveness of ketamine addition between axillary and interscalene block, since the doses and routes of administration of ketamine and ropivacaine in the two studies were exactly the same [19]. In another prospective, randomized study, Woo et al. observed no significant effect of intravenous ketamine administration on postoperative pain and total analgesic consumption in patients subjected to interscalene brachial plexus block [20].

Ketamine administration has been associated with a higher incidence of adverse effects such as dizziness, nausea, vomiting, and hallucinations as compared to tramadol [11]. Furthermore, ketamine has also been associated with a significantly higher rate of postoperative nystagmus, according to Zaman et al. [17]. According to Imbellon et al., the incidence of ketamine-induced hallucinations is dose-dependent, suggesting that a dose of 0.1 mg/kg is adequate for sedation and pain relief, whereas a dose of up to 0.5 mg/kg leads to no additional side effects [21]. However, a dose of 1.5-3.0 mg/kg may result in an increased rate of postoperative anxiety and confusion as compared to control patients [22]. According to Lee et al., intravenous use of ketamine may be associated with significantly more complications than regional use of ketamine in an interscalene block [19]. This is also a finding in the present study, since it was shown that the intravenous administration of ketamine as supplementation in the axillary block at an average calculated dose of 0.37 mg/kg (with a range of 0.25-0.55 mg/kg) resulted in a higher incidence of hallucinations in 33.3% of the patients. However, the regional administration of ketamine did not cause any such episodes. It should be noted that our study was not powered to detect the incidence of adverse effects after ketamine administration. Therefore, more definitive conclusions can only be drawn from future studies specifically powered to detect these outcomes. 

Overall, the results of the present study are in line with those of other studies. Despite ketamine's inferior blockade and analgesic properties compared to other adjuvants, its addition either intravenously or regionally still performs better than the local anesthetic alone. However, the potential for a higher incidence of hallucinations associated with the intravenous administration of ketamine could adversely affect the applicability of ketamine supplementation via the intravenous route due to a questionable risk-benefit profile, leaving more room for regional administration. Our study adds some valuable information to the considerable body of data already existing, which may contribute to the understanding of the role and safe employment of ketamine as an adjuvant in regional anesthesia or elsewhere [23], as its use is still "off-label" for several treatments.

It is acknowledged that the present study has some limitations. First, its conclusions are specific to the ropivacaine solution that was used. Therefore, more trials are required for investigating other local anesthetic solutions. Second, the dose of ketamine that was employed was the same (30 mg) and not body weight-adjusted. A fixed dose of 30 mg ketamine was selected based on previous studies investigating ketamine as an adjuvant in brachial plexus blockade, where similar absolute doses were shown to provide analgesic benefit while minimizing psychotomimetic adverse effects [15,19,21]. Fixed-dose regimens have been used to simplify administration and reduce the risk of higher cumulative exposure associated with weight-adjusted dosing, particularly in ambulatory settings. The possibility of attrition bias and performance bias exists. An additional limitation is the fact that the study was not double-blind since the principal investigator and collector of the clinical data was a trainee anesthesiologist, a member of the operating team, who was not blinded to the route of ketamine administration. However, this was partially compensated for by the fact that the investigator analyzing the data was unaware of group allocation. Furthermore, randomization was not stratified by the type of surgery, which could potentially influence pain outcomes. The study was powered to detect differences in postoperative pain scores, which constituted the primary outcome. It was not specifically powered to detect differences in secondary outcomes, including postoperative opioid consumption or adverse effects; therefore, these findings should be interpreted with caution.

## Conclusions

The present study concluded that intravenous and regional addition of ketamine to the ropivacaine solution enhances postoperative analgesia in patients undergoing axillary brachial plexus block at 16, 20, and 24 hours after surgery. These results suggest that both regional and intravenous administration of ketamine provide a stronger analgesic effect than the local anesthetic drug alone, thus attenuating rebound pain. Additionally, the administration of ketamine as an adjuvant seems to accelerate the establishment of sensory and motor block. No statistically significant difference was found in the achievement of the complete motor blockade and postoperative opioid (tramadol) intake. Intravenous administration of ketamine was also found to increase the rate of postoperative hallucinations. Further studies are needed to fully elucidate the role of ketamine addition in axillary brachial plexus block.
